# Mining core genes for low-phosphorus tolerance in barley leaves based on WGCNA and analysis of the HPT gene family

**DOI:** 10.3389/fpls.2026.1935451

**Published:** 2026-10-30

**Authors:** Panrong Ren, Run Zhang, Qi Guo, Qi Zeng, Ye Hua, Jiaojiao Jin, Qian Li, Qingshan Ma, Yiyan Ren, Yan Qiao, Julian Zhao, Fang Yang

**Affiliations:** 1 School of Agriculture and Bioengineering, Longdong University, Qingyang, China; 2 Gansu Key Laboratory of Protection and Utilization for Biological Resources and Ecological Restoration in Longdong, Longdong University, Qingyang, China

**Keywords:** gene family, *Hordeum vulgare*, HPT, low-phosphorus stress, WGCNA

## Abstract

Phosphorus deficiency is a major abiotic stress limiting crop growth, and mining low-phosphorus tolerance genes is crucial for breeding phosphorus-efficient varieties. In this study, phosphorus-efficient barley GN121 was used as material to reanalyze leaf transcriptome data under low-phosphorus stress. Differentially expressed genes (DEGs) were screened at 3, 19 and 23 days of stress, with a total of 4,043 non-redundant DEGs obtained. Weighted Gene Co-expression Network Analysis (WGCNA) divided these DEGs into 14 modules, and the MEbrown module (492 genes) was identified as the key module with extremely significant positive correlation with leaf phosphorus content (r=0.75, P = 3.2×10^-4^). *HvHPT4* encoding homogentisate phytyltransferase (HPT) was screened as the key hub gene. Genome-wide identification revealed 5 HPT family members (*HvHPT1*-*HvHPT5*) in barley, distributed on 1H, 2H and 7H chromosomes, all encoding hydrophobic transmembrane proteins with structural differentiation. Promoter cis-acting element analysis showed *HvHPT* genes contained various stress and hormone response elements, with *HvHPT3* having the most stress-related elements. Expression profiles were inspected for four abiotic-stress conditions. RT-qPCR assays in this study confirmed that *HvHPT3* and *HvHPT4* respond to low-phosphorus and drought stress. Additional expression trends for osmotic and zinc stress were obtained from the public BarleyExpDB database and were not independently validated in our wet-lab experiments. Other family members exhibited stress-specific expression patterns under the tested conditions. This study constructed the low-phosphorus stress response gene co-expression network in barley leaves, clarified the characteristics and expression patterns of the *HPT* gene family, and revealed the key role of *HvHPT3* and *HvHPT4* in abiotic stress cross-response, providing candidate gene resources and a theoretical reference for further exploring crop phosphorus-efficiency genetic improvement.

## Introduction

Phosphorus (P), an indispensable macronutrient for plant growth and development, ranks as the second essential nutrient for plants. It is involved in a variety of core physiological processes including nucleic acid synthesis, energy metabolism, cell membrane construction, signal transduction and photosynthesis, and exerts an irreplaceable regulatory effect on the entire life cycle of plants, from root establishment at the seedling stage to flowering and fruiting at the late growth stage (Lambers, 2022; Veneklaas et al., 2012). Inorganic phosphorus (Pi), which can be directly metabolized and utilized in plants, accounts for only about 15% of the total phosphorus and is mainly stored in large cellular vacuoles. Under low-phosphorus stress, plants maintain basic physiological functions through the mechanism of vacuolar phosphorus redistribution (Yang et al., 2017; Roychowdhury et al., 2023). However, phosphorus in soil is highly prone to form insoluble compounds with metal ions such as aluminum, iron and calcium, leading to low-phosphorus conditions in approximately 40% of arable land worldwide. In China, more than 80 million hectares of arable land suffers from low-phosphorus stress, with over 30% classified as severely phosphorus-deficient soil. This situation directly hinders crop growth, reduces yields and poses a potential threat to national food security (Adnan et al., 2025; Alewell et al., 2020). Meanwhile, phosphate rock is a non-renewable resource, and its global reserves can only meet mining demands for the next 50 to 100 years. Excessive application of phosphate fertilizers not only increases agricultural production costs but also triggers a series of environmental problems such as soil acidification and water eutrophication (Cooper et al., 2011; Kochian, 2012). Therefore, mining the core genes for low-phosphorus tolerance in plants by molecular biological methods, analyzing their molecular regulatory mechanisms, and further breeding phosphorus-efficient crop varieties have become the core approaches to alleviate phosphorus resource shortage and realize the green and sustainable development of agriculture.

Barley (*Hordeum vulgare* L.), the fourth largest cereal crop in the world, has multiple uses including food, forage and industrial brewing. It features a relatively simple genome, rich genetic diversity and strong stress resistance (including tolerance to low phosphorus, salt and alkali, and drought), making it an ideal model crop for studying plant stress resistance mechanisms (Mascher et al., 2017; Geng et al., 2022; Mayer et al., 2012). In previous studies, our research group successfully screened a phosphorus-efficient genotype GN121 and a phosphorus-sensitive genotype GN42 through pot and hydroponic experiments. Under low-phosphorus conditions, GN121 exhibited excellent characteristics such as well-developed roots, high phosphorus absorption efficiency and a small reduction in biomass (Ren et al., 2016). The research group further performed transcriptome sequencing on the roots and leaves of GN121 under low-phosphorus stress, and obtained a large number of differentially expressed genes (Ren et al., 2018). Leaves are the main sites of plant photosynthesis and important organs for phosphorus accumulation (accounting for about 20%-30% of the total phosphorus in plants), and they can adapt to low-phosphorus environments by regulating photosynthetic rate, phosphorus redistribution, phospholipid degradation and glycolipid replacement synthesis (Suriyagoda et al., 2023; Mo et al., 2019). For example, under low-phosphorus stress, plant leaves release inorganic phosphorus by degrading phospholipids and synthesize galactolipids and sulfolipids to replace phospholipids, thus maintaining the stability of membrane structure (Soumya et al., 2022). Meanwhile, plants can reduce phosphorus consumption by adjusting the efficiency of photosynthetic electron transport (Carstensen et al., 2018).

Traditional transcriptome analysis mainly relies on the screening of differentially expressed genes (DEGs), but this method is difficult to reveal the synergistic regulatory relationships among genes and is prone to missing key genes closely associated with traits. This results in problems such as a wide screening range and high verification costs in subsequent functional verification work (Langfelder and Horvath, 2008). Weighted Gene Co-expression Network Analysis (WGCNA) is an efficient systems biology analysis method. This method clusters genes into different modules according to the similarity of their expression patterns, and can quickly screen out key modules and key hub genes regulating target traits through correlation analysis between modules and target traits, which significantly improves the pertinence and efficiency of gene mining (Serin et al., 2016; Ren et al., 2025). At present, this method has been successfully applied to the mining of stress response genes in various crops such as rice, maize and potato. For instance, Wang et al. (2020) identified core genes in rice in response to cadmium stress using WGCNA; Zeng et al. (2022) screened key modules regulating cold tolerance in rice through this method; and Zhou et al. (2023) analyzed the gene network related to low-phosphorus stress in soybean with the help of WGCNA. In addition, in the study on low-phosphorus tolerance of rapeseed, WGCNA has been used to successfully screen core genes related to phosphorus absorption efficiency (Zulfiqar et al., 2025); in maize research, hub genes regulating phosphorus metabolism have also been identified via this method (Hao et al., 2025).

Homogentisate phytyltransferase (HPT) belongs to the UbiA superfamily of isopentenyltransferases and is a key rate-limiting enzyme in the biosynthetic pathway of plant vitamin E (tocopherols). Its catalytic activity directly determines the total content and accumulation efficiency of vitamin E in plants (Nowicka and Kruk, 2016; Morelli et al., 2023). As an important fat-soluble antioxidant, vitamin E can scavenge reactive oxygen species (ROS) and protect biological membranes from oxidative damage, playing a crucial role in plant responses to abiotic stresses such as drought, salt stress and low temperature (Faizan et al., 2023; Miret and Munné-Bosch, 2015; Kreszies et al., 2025). Previous studies have shown that the overexpression of the Arabidopsis AtHPT gene can increase the vitamin E content in plant leaves, thereby enhancing plant stress resistance (Savidge et al., 2002; Collakova and DellaPenna, 2003). The germination rate of rice lines with HPT gene silencing is significantly reduced and the proportion of abnormal seedlings is increased, indicating that HPT is an important guarantee for rice seeds to maintain normal germination, improve stress resistance and prolong seed longevity (Chen et al., 2016). In addition, studies have found that the transcriptional level of HPT in the peel of olive fruits is regulated by water status, temperature, light and damage treatment, suggesting that it may be involved in the response to abiotic stresses (Narváez et al., 2025). RNAi-mediated HPT-silenced transgenic tobacco plants show a significant increase in sensitivity to salt stress and sorbitol stress (Abbasi et al., 2007). Zeng et al. (2023) found that two HPT genes in barley (HvHPT1-HORVU.MOREX.r3.7HG0740290.1; HvHPT2-HORVU.MOREX.r3.2HG0208140.1) are widely expressed in various plant tissues. Moreover, the prediction of promoter cis-acting elements shows that both HvHPT3 and HvHPT4 contain conserved elements related to light, plant hormones, low temperature, drought and defense responses, and various stresses can induce the up-regulation of HvHPT3 and HvHPT4 expression. Although considerable progress has been made in the research on HPT genes, genome-wide identification of the HPT gene family in barley (*Hordeum vulgare*) has not been reported, and its role in the cross-response of barley to abiotic stresses remains to be further elucidated.

Based on the above research background, this study took the phosphorus-efficient barley GN121 as the experimental material and reanalyzed the leaf sequencing results in the 2018 transcriptome data. Through the screening of differentially expressed genes, WGCNA analysis, core gene identification and systematic analysis of the HPT gene family, this study aimed to achieve two objectives: (1) construct a gene co-expression network for the response of barley leaves to low-phosphorus stress and mine the key hub genes of leaves in response to low-phosphorus stress; (2) analyze the evolutionary characteristics and gene structure of the HPT gene family and clarify the functions of core genes under various abiotic stresses. The results of this study will improve the molecular regulatory network of low-phosphorus tolerance in barley, reveal the stress resistance function of HPT genes, and provide candidate gene resources and theoretical reference for subsequent research on crop phosphorus-efficiency genetic improvement.

## Materials and methods

### Data sources

The original transcriptome data were obtained from a study published by our team in 2018 (NCBI Accession No.: SRP131833), which was sequenced on the Illumina HiSeq X 10 platform with a paired-end 150 bp sequencing strategy (Ren et al., 2018). Leaf inorganic-phosphorus concentration values used for module-trait correlation in this study were also sourced from Ren et al. (2018). Identical final processed numerical values from that prior publication were directly reused in the present WGCNA analysis. Raw spectrophotometric measurement files of phosphorus assays were not re-extracted or re-analyzed in the current manuscript. In the original work, leaf inorganic phosphorus content was determined by the molybdenum-antimony colorimetric method (Kowalenko and Babuin, 2007).

### Screening of differentially expressed genes

FastQC software (v0.11.9) was used to evaluate the quality of the original transcriptome data, with evaluation indicators including base quality values (Q20, Q30), GC content and adapter contamination. Trimmomatic software (v0.39) was employed for data filtering to remove sequences containing adapter sequences, low-quality bases and overly short sequences, so as to obtain high-quality clean data.

The barley reference genome (*Hordeum vulgare* L.) and annotation files were downloaded from the Ensembl Plants database (https://plants.ensembl.org/). Hisat2 software (v2.2.10) was used to construct a genome index with the --dta parameter to support subsequent transcript assembly, and the clean data were aligned to the reference genome with default alignment parameters. Samtools software (v1.17) was utilized to convert the alignment results (SAM files) into BAM files, sort them and remove duplicate sequences. Stringtie software (v1.3.3) was adopted for transcript assembly under the guidance of reference genome annotation, and the FPKM (Fragments Per Kilobase of exon model per Million mapped fragments) value was used to represent the gene expression level.

DESeq2 software (v3.11) was used for differential expression analysis. First, the gene expression matrix was standardized, and then statistical tests were performed based on the negative binomial distribution model. The screening criteria were set as follows: adjusted P value (Padj) < 0.05 and |log_2_(fold change)| ≥ 0.5. Differentially expressed genes (DEGs) on the 3rd, 19th and 23rd days were identified respectively, and the VennDiagram package of R language was used to draw a Venn diagram to obtain the union of the three groups as the gene set for subsequent analysis.

### Construction of weighted gene co-expression network

The WGCNA package of TBtools software was used to perform co-expression network analysis on the union of differentially expressed genes, with the specific steps as follows: (1) Data preprocessing: First, genes with FPKM < 1 and expressed in less than 90% of the samples were filtered out to retain stably expressed genes. Then, outlier samples were detected through sample cluster analysis (hclust function) and correlation analysis (cor function), and the dynamic tree cutting method was used to eliminate outlier samples to ensure the reliability of network construction. Notably, the input gene set for WGCNA is the union of DEGs derived from three experimental contrasts: 3-day low-phosphorus stress, 19-day sustained low-phosphorus stress, and 23-day phosphorus-recovery (Pi-resupply) treatment. Therefore, the resulting co-expression network integrates transcriptional responses to both phosphorus starvation and physiological reconstruction during phosphorus re-supply, rather than only genes responding to continuous low-phosphorus stress. (2) Soft threshold screening: Based on the scale-free topology model, the range of soft threshold (power) was set to 1~30. The scale-free network fitting index (R^2^) and average connectivity corresponding to each power value were calculated, and the minimum power value with R^2^> 0.7 and reaching the plateau was selected to construct the network. The final power value determined in this study was 7. (3) Module division: The dynamic tree cutting algorithm was adopted with the minimum module size (minModuleSize) set to 30, the pruning height (cutreeDynamic.cutHeight) set to 0.25 and the mergeCutHeight set to 0.25. Genes with similar expression patterns were divided into different modules, and each module was marked with a different color. (4) Module-trait association: The module eigengene (ME) value of each module was calculated, which represents the overall expression level of the module. Pearson correlation analysis was used to calculate the correlation coefficient and significance between the ME value and the leaf phosphorus content trait, so as to screen out the key modules significantly positively correlated with phosphorus content (P < 0.001). Leaf inorganic-phosphorus concentration was phenotyped for each biological replicate plant that provided tissue for RNA-seq library construction (Ren et al., 2018). Attempts to import raw per-replicate phosphorus measurements directly into the WGCNA module-trait workflow resulted in computational runtime errors. To resolve this issue, the arithmetic mean phosphorus-content value of each treatment-time-point group was calculated, and this group-average value was assigned to every RNA-seq biological replicate belonging to that treatment. (5) Core gene screening: The gene significance (GS) and module membership (MM) of each gene in the module were calculated. GS was defined as the absolute value of the correlation between gene expression level and phosphorus content, and MM was defined as the absolute value of the correlation between gene expression level and ME value. Genes with GS > 0.5 and MM > 0.8 were screened as key hub genes to ensure that the core genes are not only closely related to the target trait but also have high connectivity in the module.

### Visualization of core genes

Cytoscape software (v3.10.1) was used to construct the gene interaction network of the key module. The gene adjacency matrix obtained from WGCNA analysis was imported, the node size was set to represent gene connectivity, the node color was set to represent gene significance, and the edge thickness was set to represent the intensity of interaction between genes. The CytoHubba plug-in (v0.1) was applied for hub-gene ranking. Three centrality metrics including degree centrality, betweenness centrality, and closeness centrality were calculated. Genes were ranked by the sum of ranks across these three metrics (comprehensive combined rank). The top-20 genes with the highest comprehensive combined rank were selected for network visualization; no additional cut-off thresholds were applied beyond this combined ranking rule.

### Identification and analysis of the HPT gene family

According to the visualization analysis results of Cytoscape, the top-ranked core gene HPT was selected for genome-wide identification and analysis combined with bioinformatics tools, with the specific steps as follows: (1) Identification of family members: The protein sequences of Arabidopsis AtHPT1 (AT2G18950) and AtHPT2 (AT3G11945) were downloaded from the TAIR database (https://www.arabidopsis.org/) as query sequences, and the barley reference genome was aligned using the BLASTP tool (E-value < 1e-5, query coverage ≥50 %, subject coverage ≥50 %) to obtain candidate HPT genes. The hidden Markov model (HMM) of the HPT conserved domain (PF03463) was downloaded from the InterPro database (https://www.ebi.ac.uk/interpro/), and HMMER software (v3.3.2) was used to verify the domain of candidate genes with the inclusion threshold set to -E 1e-3; sequences with domain bit-score <20 were discarded. Sequences without complete HPT conserved domains were removed, and redundant and incompletely annotated genes were manually eliminated to finally determine the members of the barley HPT gene family. (2) Chromosomal localization: The information on chromosome number, start position and end position of HPT family members was extracted from the barley genome annotation file, and the “Gene Location Visualize” function of TBtools software (v2.034) was used to draw a chromosomal localization map to clarify the distribution position of each member on the chromosome. (3) Conserved sequence alignment analysis: ClustalX software (v2.1) was used to perform multiple sequence alignment on the HPT protein sequences of barley and Arabidopsis with default parameters. (4) Analysis of gene structure and conserved motifs: The “Gene Structure View (Advanced)” function of TBtools software was used to extract the information on exons, introns and UTR regions of HPT genes from the genome annotation file and draw a schematic diagram of gene structure. Conserved motifs were predicted through the MEME website (http://meme-suite.org/tools/meme) with the parameters set as follows: maximum number of motifs was 10, motif length range was 6–50 aa, and other parameters were default values. TBtools software was used to integrate the analysis results of gene structure and conserved motifs. (5) Analysis of cis-acting elements: The 2000 bp promoter sequence upstream of the start codon of HPT genes was extracted from the barley genome using TBtools software and submitted to the PlantCARE website (https://bioinformatics.psb.ugent.be/webtools/plantcare/html/) for cis-acting element prediction. All putative cis-elements returned by PlantCARE were retained without additional score-based filtering; only element functional annotation was used for manual classification, and low-complexity simple repeat motifs were excluded manually. The prediction results were classified and counted, including abiotic stress response elements and hormone response elements, and TBtools software was used to draw a heat map of cis-acting element distribution.

### Real-time quantitative PCR

BarleyExpDB (http://barleyexp.com/index.html) was only used for preliminary reference collection of HvHPT family expression background, and was not adopted as core experimental dataset in this study. To validate the expression patterns of five *HvHPT* genes under low-phosphorus and drought stresses observed in the original published RNA-seq dataset (SRP131833, Ren et al., 2018), seedlings of the phosphorus-efficient barley genotype GN121 were used as experimental materials. This RT-qPCR represents an independent biological experiment: fresh plant materials were newly cultivated and sampled in our laboratory, rather than re-using stored RNA samples from the 2018 transcriptome study. Seedlings under low-phosphorus treatment were cultured following the method described by Ren et al. (2018). Briefly, plants were grown in a controlled-environment growth chamber with 16 h light/8 h dark photoperiod, temperature set to 20 ± 5 °C, relative humidity maintained at 50–70 %, and photosynthetic photon flux density of 300 µmol photons/m^2^/s^1^. The normal-phosphorus control nutrient solution contained 0.39 mM KH_2_PO_4_; the low-phosphorus treatment solution contained 0.039 mM KH_2_PO_4_, and potassium levels were balanced by addition of K_2_SO_4_. For phosphorus-related treatments, leaf samples were collected at 19 d under sustained low-phosphorus stress. For drought-stress treatment: 12-day-old uniformly-grown barley seedlings cultivated in complete nutrient solution were subjected to stress by complete withholding of nutrient solution for 10 consecutive days before leaf sampling. It should be noted that this experimental setup introduces not only water deficit but also simultaneous nutrient deprivation, because no nutrient solution was supplied throughout the drought period. Leaf relative water content (RWC) or other physiological markers reflecting drought severity were not measured in this independent RT-qPCR assay. Untreated seedlings were collected as mock controls. Fresh leaf samples from each treatment were immediately snap-frozen in liquid nitrogen and ground thoroughly into fine powder. Total RNA was isolated using TRIzol reagent (Invitrogen, Cat. No. 15596026). A total of 1.5 μg intact total RNA was reverse-transcribed into first-strand cDNA with the PrimeScript^®^ RT reagent Kit (TaKaRa, Dalian). Diluted cDNA samples served as templates for RT-qPCR assays, and three independent biological replicates were included for every treatment group. The RT-qPCR thermal profile was configured as follows: an initial pre-denaturation at 95 °C for 30 s, followed by 40 cycles of 95 °C for 5 s and combined annealing-extension at 56 °C for 30 s. Melting curve analysis was conducted post-amplification to confirm primer specificity, with the program run at 95 °C for 15 s, 56 °C for 30 s, and 95 °C for 15 s. The housekeeping gene *HvActin* was adopted as the internal reference for normalization of target gene expression. Relative expression levels of *HvHPT1~HvHPT5* were calculated using the 2^−ΔΔCt^ method. All specific RT-qPCR primers targeting the five *HvHPT* genes were designed via the NCBI Primer-BLAST online tool, and all primer sequences are provided in **Supplementary Table S1**.

### Data analysis

All experimental data were statistically analyzed using SPSS 26.0 software. Independent samples t-test was used for the test of significant differences, one-way analysis of variance (ANOVA) was used for comparison among multiple groups, and Duncan’s new multiple range method was used for multiple comparisons. P < 0.05 indicated a significant difference. Excel (v16.78), TBtools (v2.034) and Cytoscape (v3.10.1) software were used for drawing charts and figures.

## Results

### Analysis of differentially expressed genes in barley leaves under low-phosphorus stress

Differential expression analysis was performed on the transcriptome data of barley leaves under low-phosphorus stress at different stages, and the results showed (**Figure 1**): a total of 154 differentially expressed genes were screened on the 3rd day of low-phosphorus stress, including 89 up-regulated genes and 65 down-regulated genes (**Figure 1A**), indicating that the number of responsive genes in barley leaves was small under short-term low-phosphorus stress, with only the basic stress response mechanism being activated. The number of differentially expressed genes increased markedly on the 19th day, with a total of 1990 identified, including 1223 up-regulated genes and 767 down-regulated genes (**Figure 1B**), suggesting that under Pi-resupply condition, barley activated the expression regulation of a large number of genes to adapt to the stress environment. After resuming normal phosphorus supply on the 23rd day, the number of differentially expressed genes further increased to 2686, including 1471 up-regulated genes and 1215 down-regulated genes (**Figure 1C**), which indicated that barley leaves completed the recovery and reconstruction of physiological state by regulating the expression of a large number of genes after the resumption of normal phosphorus supply. It should be highlighted that DEGs obtained at 23 d originate from the phosphorus-recovery (Pi-resupply) condition, rather than continuous low-phosphorus stress. These genes represent transcriptional programmes for physiological readjustment upon phosphorus repletion, and were pooled together with DEGs from 3 d and 19 d low-phosphorus starvation for subsequent WGCNA analysis. Venn diagram analysis showed that there were only 14 differentially expressed genes common to the three stages, while the union of the three contained 4043 non-redundant differentially expressed genes (**Figure 1D**), demonstrating that the response of barley leaves to low-phosphorus stress had obvious time specificity, and there were significant differences in the regulatory gene pools at different stress stages. In addition, the number of genes involved in the response increased significantly with the extension of stress time, implying that plants would activate a more extensive molecular regulatory network to adapt to the stress environment under Pi-resupply condition.

**Figure 1:**
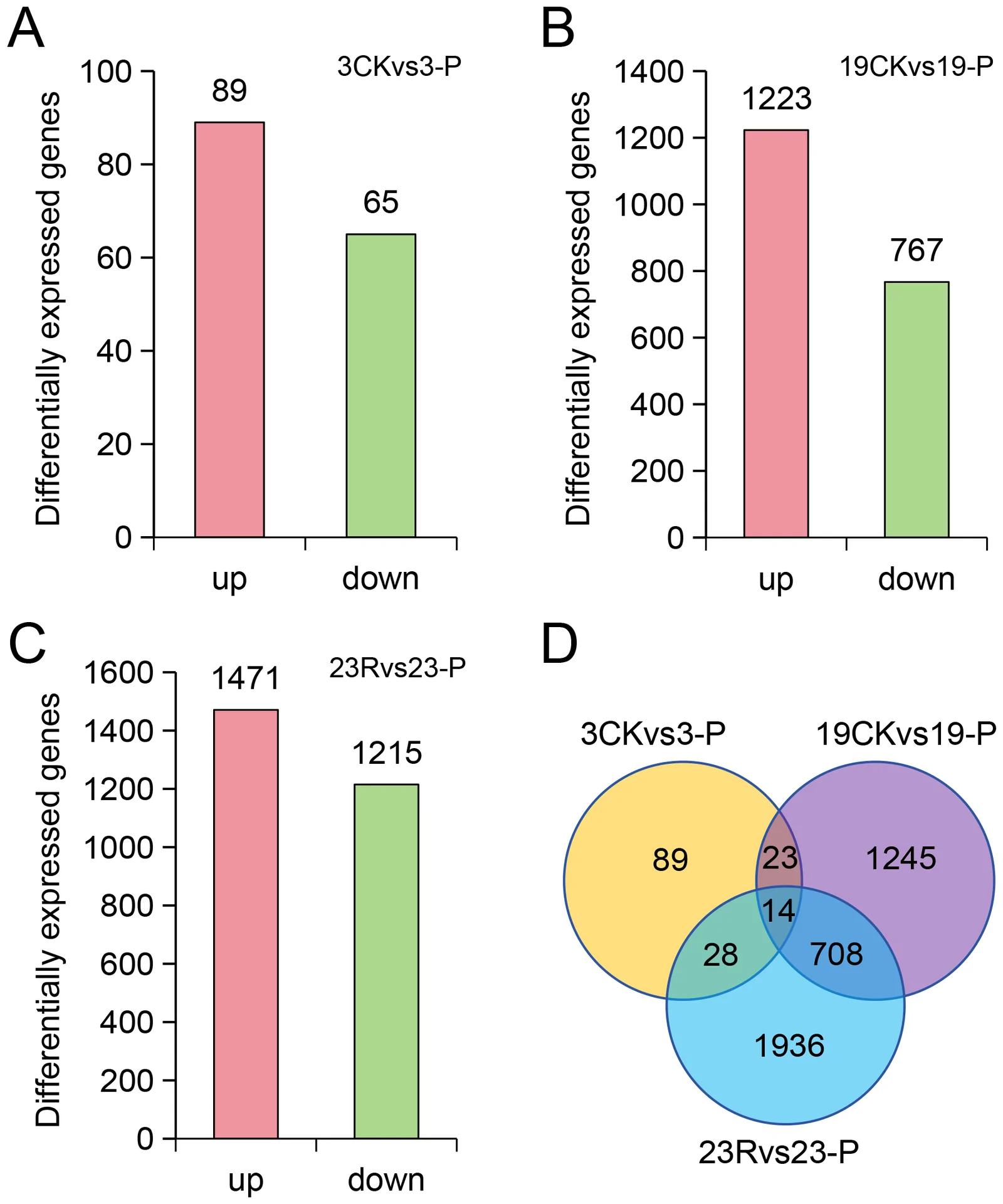
Number of differentially expressed genes in barley leaves under low-phosphorus stress at different stages. **(A-C)** show the statistics of the number of differentially expressed genes on the 3rd, 19th and 23rd days, respectively, with red representing up-regulated genes and green representing down-regulated genes; **(D)** is the Venn diagram of differentially expressed genes at the three time points.

### Construction of the co-expression network of barley leaves in response to low-phosphorus stress (WGCNA)

WGCNA analysis was performed on 4043 non-redundant differentially expressed genes, which combined DEGs from 3-day and 19-day low-phosphorus starvation as well as 23-day Pi-resupply treatment. First, soft threshold screening was carried out to construct a gene co-expression network conforming to the characteristics of the scale-free network, and the scale-free network fitting degree is the core index to measure the reliability of network construction. The results showed that with the increase of soft threshold (power), the fitting degree (R²) of the scale-free network model increased gradually and tended to be stable, while the average connectivity decreased gradually (**Figure 2**). When the soft threshold (power) = 7, the fitting degree R² of the scale-free network model was 0.72 and the slope was -0.77, which met the core requirements for scale-free network construction (**Supplementary Figure S1**). Therefore, power = 7 was selected for subsequent co-expression network construction. The dynamic tree cutting algorithm was used with the minimum module size set to 30 and the pruning height set to 0.25, and genes with similar expression patterns were divided into 14 co-expression modules, each marked with a different color (**Supplementary Figure S2**). There were significant differences in the gene number distribution of each module: the turquoise module had the largest number of genes (426), accounting for 14.86% of the total number of genes; the grey module had the smallest number of genes (32), accounting for 1.12%; the number of genes in the other modules ranged from 50 to 350, indicating that differentially expressed genes could form regulatory modules of different scales according to their expression patterns (**Supplementary Table S2**).

**Figure 2:**
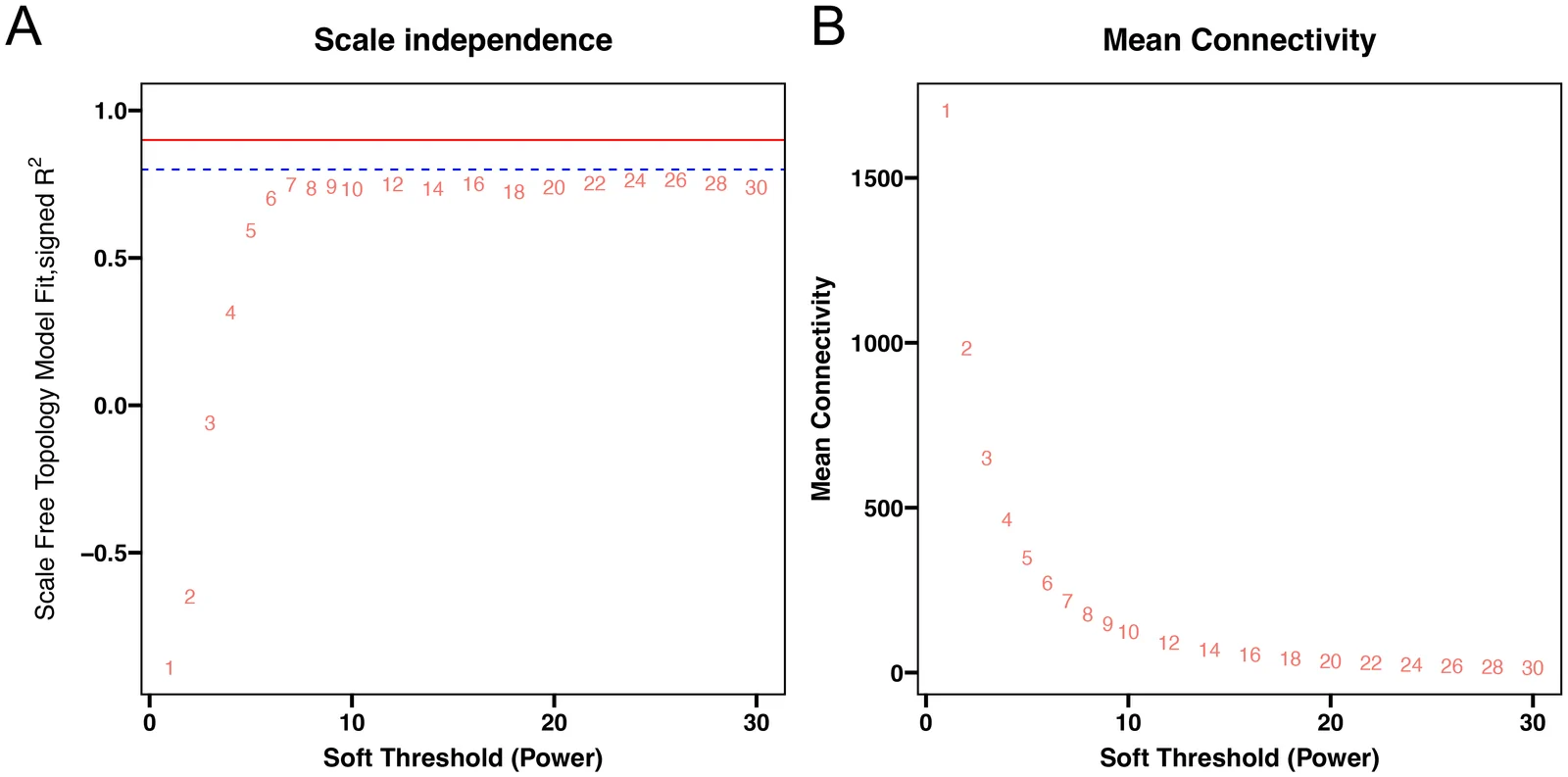
Soft threshold screening in WGCNA analysis. **(A)** shows the relationship between soft threshold and scale-free network fitting degree; **(B)** shows the relationship between soft threshold and average connectivity.

### Correlation analysis between modules and phosphorus content

To screen the key modules closely related to the response to low-phosphorus stress, Pearson correlation analysis was performed between the module eigengene (ME) of the 14 co-expression modules (representing the overall expression level of the module) and phosphorus content, and the significance of the correlation was calculated (**Figure 3**). The results showed that there were significant differences in the correlation degree between different modules and leaf phosphorus content: the MEbrown module was extremely significantly positively correlated with phosphorus content (r = 0.75, P = 3.2×10^-4^), the MEred module was extremely significantly negatively correlated with phosphorus content (r = -0.82, P = 3.7×10^-5^), the MEpurple module was significantly negatively correlated (r = -0.61, P = 0.0074), and the MEyellow module was significantly positively correlated (r = 0.53, P = 0.024). The correlation between the other modules and phosphorus content did not reach a significant level. To screen the positive regulatory genes for low-phosphorus stress, the MEbrown module was identified as the key module of barley leaves in response to low-phosphorus stress in this study, which contained 492 genes.

**Figure 3:**
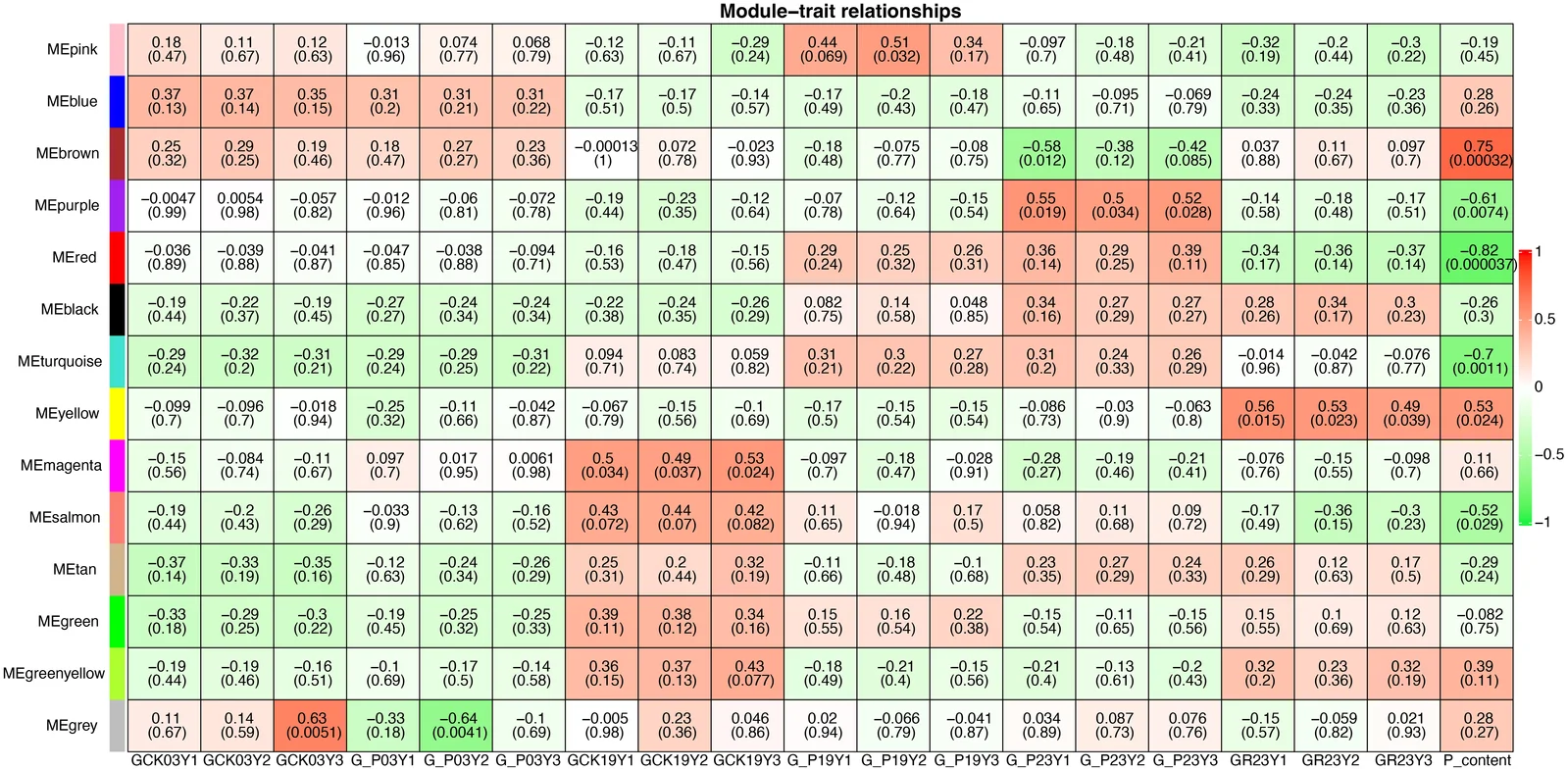
Heat-map of module-eigengene-sample and module-trait correlations. The columns on the left side show module eigengene expression for each individual RNA-seq biological sample. The far-right column labelled P_content represents the leaf inorganic-phosphorus phenotypic trait. Each cell displays the Pearson correlation coefficient and corresponding P-value (within parentheses). Colour intensity corresponds to correlation magnitude; red denotes positive correlation, green denotes negative correlation.

### Screening and visualization of key hub genes

To obtain core genes, Cytoscape software was used to visualize the regulatory network of the MEbrown module. The CytoHubba plug-in was employed to calculate the degree centrality, betweenness centrality and closeness centrality of genes, and the top 20 genes with comprehensive ranking were screened out to draw a network diagram. In this study, the top-ranked gene HORVU.MOREX.r3.1HG0080260 was selected as the core candidate gene in response to low-phosphorus stress, which encodes homogentisate phytyltransferase (**Figure 4**).

**Figure 4:**
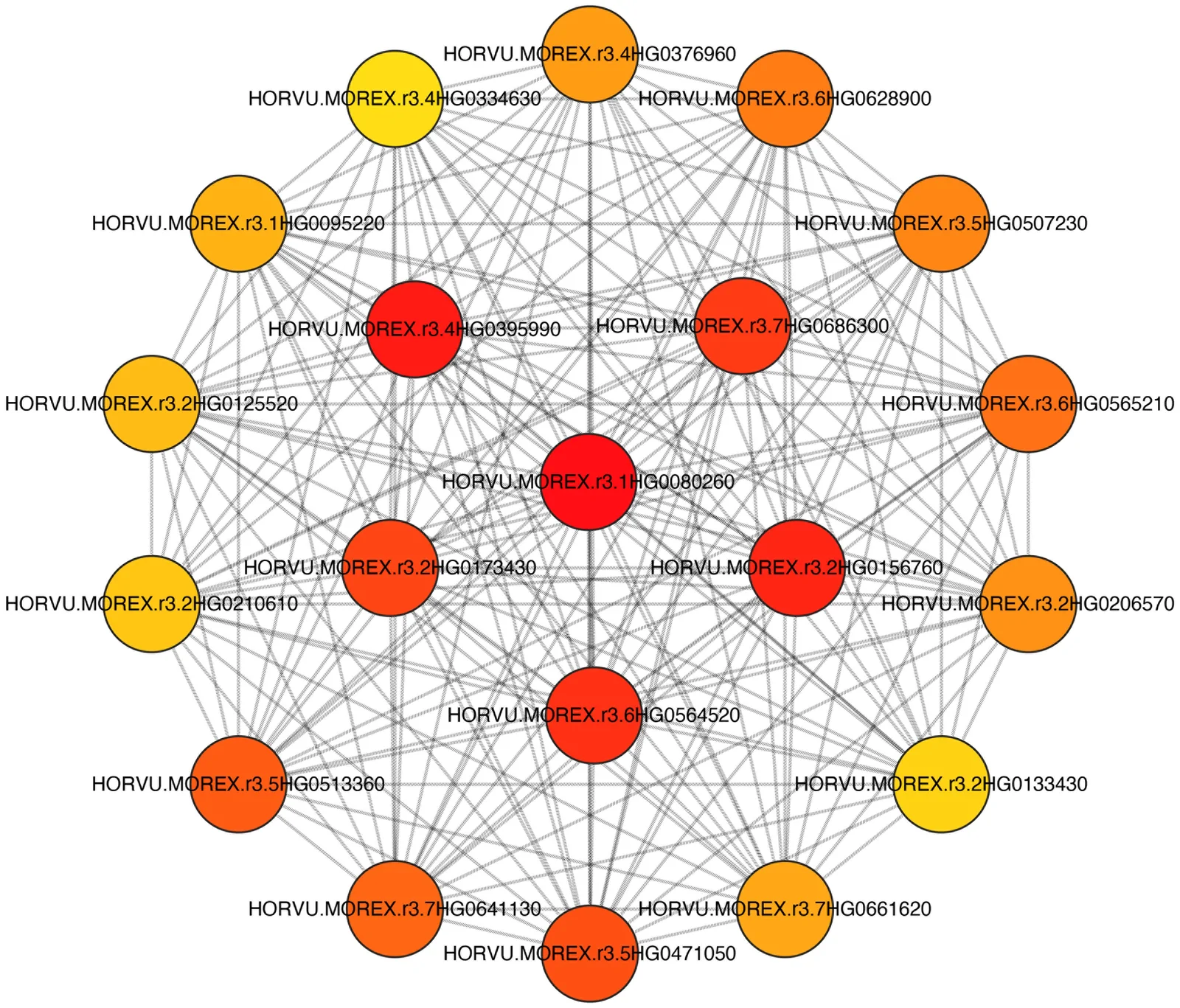
Visualization of the MEbrown module gene-interaction network. Node colour positively corresponds to the comprehensive hub-gene rank calculated by the CytoHubba plug-in, which integrates three centrality metrics: degree centrality, betweenness centrality, and closeness centrality. darker-red nodes indicate higher-ranked hub genes. The centrally highlighted red node represents the top-ranked hub gene HORVU.MOREX.r3.1HG0080260 (*HvHPT4*). Edge thickness indicates the strength of pairwise gene-to-gene co-expression connectivity.

### Genome-wide identification and analysis of the barley HPT gene family

Taking the key hub gene *HORVU.MOREX.r3.1HG0080260* (homogentisate phytyltransferase gene) as the research object, we note that multiple high-ranked hub genes were retrieved from the MEbrown co-expression network. We selected *HvHPT4* and its corresponding gene family for comprehensive genome-wide characterization for the following reasons. First, *HvHPT4* encodes a rate-limiting enzyme for vitamin E (tocopherol) biosynthesis, and vitamin-E-mediated antioxidant protection is physiologically highly relevant to leaf chloroplast damage and reactive oxygen species accumulation triggered by low-phosphorus stress. Second, a systematic genome-wide survey of the barley *HPT* gene family had not been published previously, while many other top-ranked hub genes in this network belong to well-characterized gene families (such as phosphate transporters and known stress-related transcription factors) that have been extensively studied in cereals under phosphorus deficiency. Third, both transcriptome and RT-qPCR assays indicated that *HvHPT4* and its paralog *HvHPT3* showed prominent stress-responsive expression patterns under abiotic stresses, pointing to a potential unreported functional connection between tocopherol metabolism and barley low-phosphorus adaptation. Genome-wide identification and systematic analysis of the barley HPT gene family were carried out to lay a foundation for analyzing the function of the HvHPT gene. Using the protein sequences of Arabidopsis AtHPT1 (*AT2G18950*) and AtHPT2 (*AT3G11945*) as reference sequences, the barley genome was aligned by the BLASTP program (E-value < 1e-5). Combined with the verification of the HPT conserved domain in the InterPro database (PF03463), candidate sequences without complete HPT conserved domains, redundant sequences and incompletely annotated sequences were eliminated. A total of 5 members of the barley HPT gene family were identified and named HvHPT1~HvHPT5 in the order of chromosomal localization. The results of chromosomal localization showed (**Figure 5**) that the 5 HPT family members were distributed on chromosomes 1H, 2H and 7H of barley without tandem repeat clustering. Among them, both HvHPT3 and HvHPT4 were localized on chromosome 1H, HvHPT2 on chromosome 2H, and HvHPT1 and HvHPT5 on chromosome 7H. Further chromosomal synteny analysis of the HPT gene family between barley and Arabidopsis found (**Supplementary Figure S22**) that all the 5 HPT genes in barley had a syntenic relationship with the 2 HPT genes in Arabidopsis.

**Figure 5:**
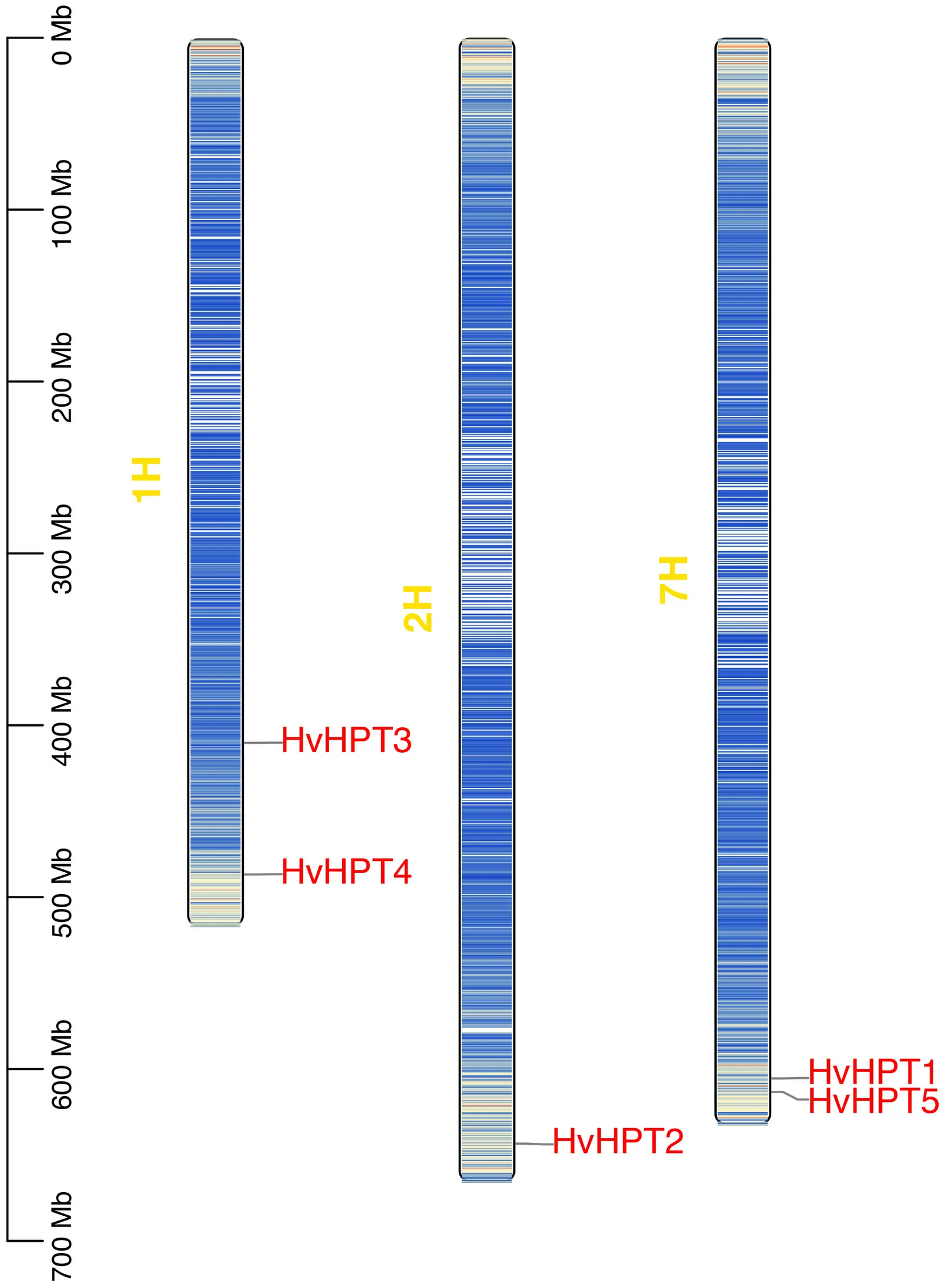
Chromosomal localization of HPT family members in barley. The black lines on the chromosomes represent gene positions, and the labels on the right-hand side of each chromosome indicate corresponding gene names.

Comprehensive analysis of the physicochemical properties of the barley HPT family members (**Supplementary Table S3**) showed the following results: the number of amino acids of the 5 HvHPT proteins ranged from 377 to 408, among which HvHPT5 had the largest number of amino acids (408) and HvHPT3 the smallest (377); the molecular weight was 40.69~45.09 kDa, and the isoelectric point (pI) was 7.59~10.08. HvHPT4 had the highest isoelectric point (10.08) and was a strongly basic protein, while the other members were weakly basic proteins; the instability index was 31.22~49.40, HvHPT3 had the lowest instability index (31.22) and was a stable protein, while HvHPT4 and HvHPT1 had an instability index exceeding 40 and were unstable proteins; the aliphatic amino acid index was 103.11~110.75, and the grand average of hydropathicity (GRAVY) was positive, ranging from 0.367 to 0.561, indicating that all HvHPT proteins were hydrophobic proteins with strong hydrophobicity; the number of transmembrane helices (TMHs) was 5~8, and none of them had signal peptides, suggesting that HvHPT proteins were transmembrane proteins localized on biological membranes.

### Analysis of gene structure and conserved motifs

The diversity of gene structure is an important basis for gene functional differentiation. Analysis of the exon-intron structure of the 5 barley HPT family members (**Figure 6A**) showed that there were certain differences in the gene structure of the barley HPT family members: all genes except HvHPT2 contained 3’ and 5’ UTR regions, and the 5’ UTR region was longer; all members contained multiple exons, among which *HvHPT4* had the least number of exons (10) and HvHPT3 the most (15). Sequence alignment between barley HPT genes and Arabidopsis HPT genes found that all HvHPT genes contained two aspartic acid-rich conserved motifs: DX [D/E] XD and DXXDXXXD (marked with a red background in **Supplementary Figure S4**), which were widely conserved in the UbiA superfamily. In addition, conserved domain analysis of the 5 HvHPT family members showed that they all contained the homogentisate phytyltransferase domain (**Supplementary Figure S5**), and the domain sequence lengths of each family member showed obvious interval differences. This indicated that the barley HPT gene family had differentiation characteristics in the core functional domain, and the length difference of the domain sequence might directly affect the composition of the catalytic active center, substrate binding ability and functional specificity of HvHPT proteins.

**Figure 6:**
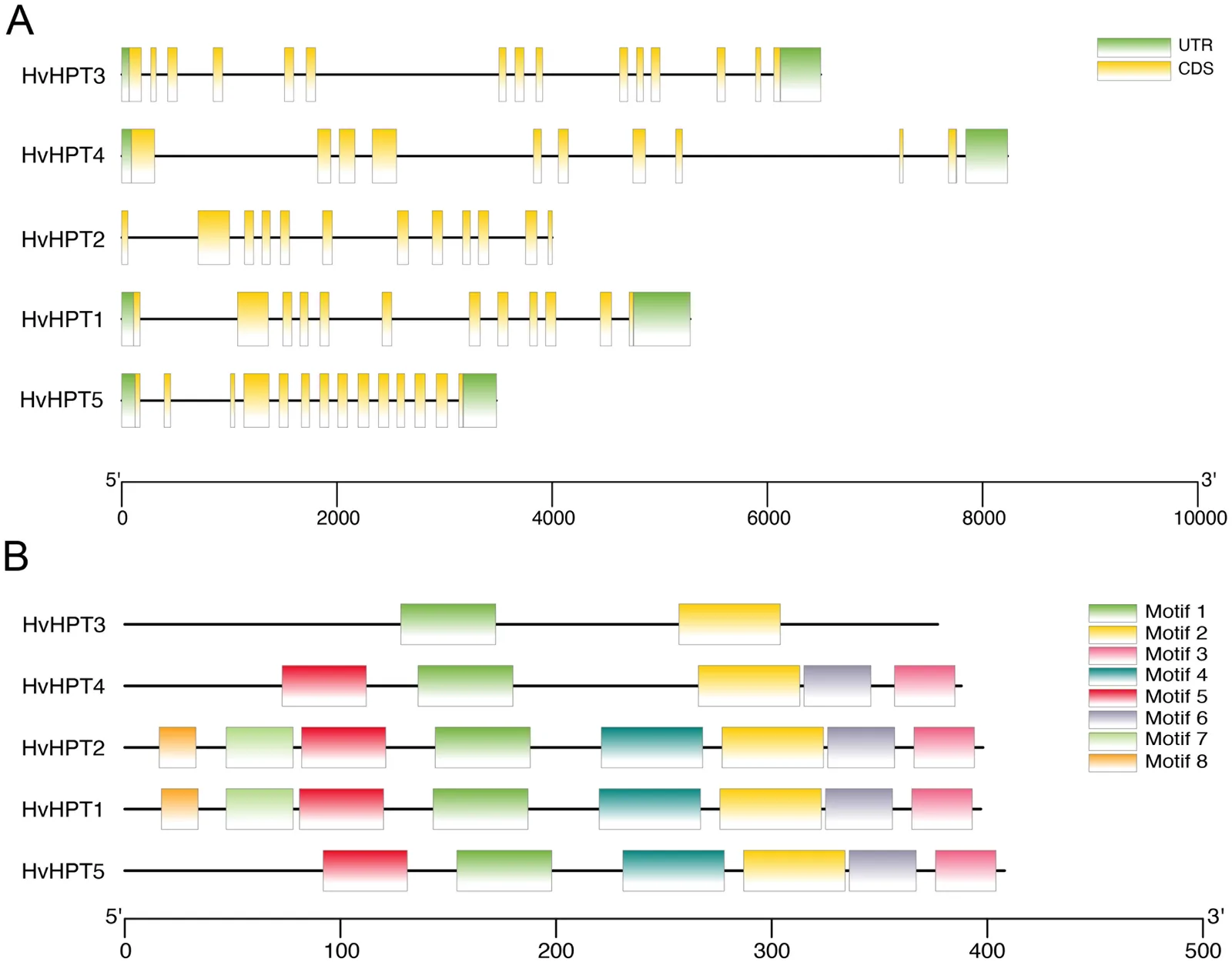
Analysis of gene structure and conserved motifs of the HPT gene family in barley. **(A)** Gene-structure diagram: black lines represent introns, yellow boxes represent exons, green boxes represent UTR regions; abscissa shows sequence length in bp. **(B)** Conserved-motif distribution diagram; different-coloured boxes denote distinct conserved motifs; abscissa shows protein-sequence length in amino acids (aa).

The MEME website was used to predict the conserved motifs of barley HPT proteins with the maximum number of motifs set to 10. The results showed that the 5 HvHPT proteins contained a total of 8 conserved motifs (Motif 1~Motif 8) (**Figure 6B**). Motif 1 and Motif 2 were core motifs common to all HvHPT family members, distributed in the middle region of the protein sequence, suggesting that these core motifs were the key parts constituting the catalytic domain of HPT proteins and were closely related to the core functions of HPT proteins. Motif 3, Motif 5 and Motif 6 existed in HvHPT4~HvHPT5, Motif 4 existed in HvHPT2~HvHPT5, and Motif 7 and Motif 8 were specific motifs of HvHPT2 and HvHPT1.

### Analysis of promoter cis-acting elements

Cis-acting elements in the promoter region are important elements for gene expression regulation, which determine the expression pattern and response characteristics of genes. In this study, the 2000 bp promoter sequence upstream of the start codon of the 5 HvHPT genes was extracted, and cis-acting element prediction and analysis were performed through the PlantCARE website (**Supplementary Figure S6**). The results showed that there were significant differences in the composition of promoter cis-acting elements of different HvHPT genes: the promoter region of HvHPT3 contained stress-related elements such as defense and stress response, drought induction and anaerobic induction, suggesting that the HvHPT3 gene might be involved in the response to various abiotic stresses; the promoter regions of HvHPT4, HvHPT2 and HvHPT1 were rich in hormone response elements, implying that their expression was regulated by abscisic acid, methyl jasmonate, salicylic acid and other hormones; the promoter region of HvHPT5 mostly contained anaerobic induction-related elements. The diversity of promoter cis-acting elements indicated that the expression regulation patterns of the barley HPT gene family were diverse, and the family genes could participate in the growth and development and stress response of barley through different regulatory pathways.

### Analysis of the expression pattern of the HvHPT gene family

To clarify the expression characteristics of the barley HvHPT gene family under abiotic stresses, we analyzed the expression patterns of *HvHPT1*~*HvHPT5* under low-phosphorus and drought stresses based on RNA-seq data, and further validated these expression profiles via RT-qPCR (**Figure 7**). Combined transcriptome and RT-qPCR results revealed that the expression of barley HvHPT family members exhibited obvious gene specificity and stress specificity under low-phosphorus and drought treatments. Transcriptome data showed that the FPKM value of *HvHPT3* increased from 37.43 in mock to 59.10 under drought stress, with an upregulation ratio of 57.9%. RT-qPCR results were consistent with this trend. Under low-phosphorus treatment, the FPKM value of HvHPT3 decreased slightly from 30.34 (mock) to 28.41. *HvHPT4* was upregulated under both low-phosphorus and drought stresses, acting as a key gene for barley to cope with these two abiotic stresses. For low-phosphorus stress, the FPKM value of *HvHPT4* rose from 54.37 (mock) to 56.63, and its RT-qPCR relative expression level was 1.75 times that of the mock group. Under drought stress, its FPKM value increased from 12.10 to 17.59. No transcripts of *HvHPT2* were detected in mock and stress groups of both treatments, suggesting that *HvHPT2* did not participate in barley responses to low-phosphorus and drought stresses. HvHPT1 was specifically induced only by drought stress rather than low-phosphorus stress. Under drought stress, the FPKM value of *HvHPT1* increased from 44.52 (mock) to 76.67, and its RT-qPCR relative expression level was up to 2.14-fold of the control. *HvHPT5* showed no detectable expression under either low-phosphorus or drought stress. Trace FPKM value (0.02) was only observed in the mock group of low-phosphorus treatment, whereas the low-phosphorus stress group and all drought-related groups had zero FPKM values. In general, the expression trends quantified by RT-qPCR were highly consistent with the FPKM results of RNA-seq, which verified the reliability of transcriptome data and clarified the specific response characteristics of the HvHPT gene family under low-phosphorus and drought stresses.

**Figure 7:**
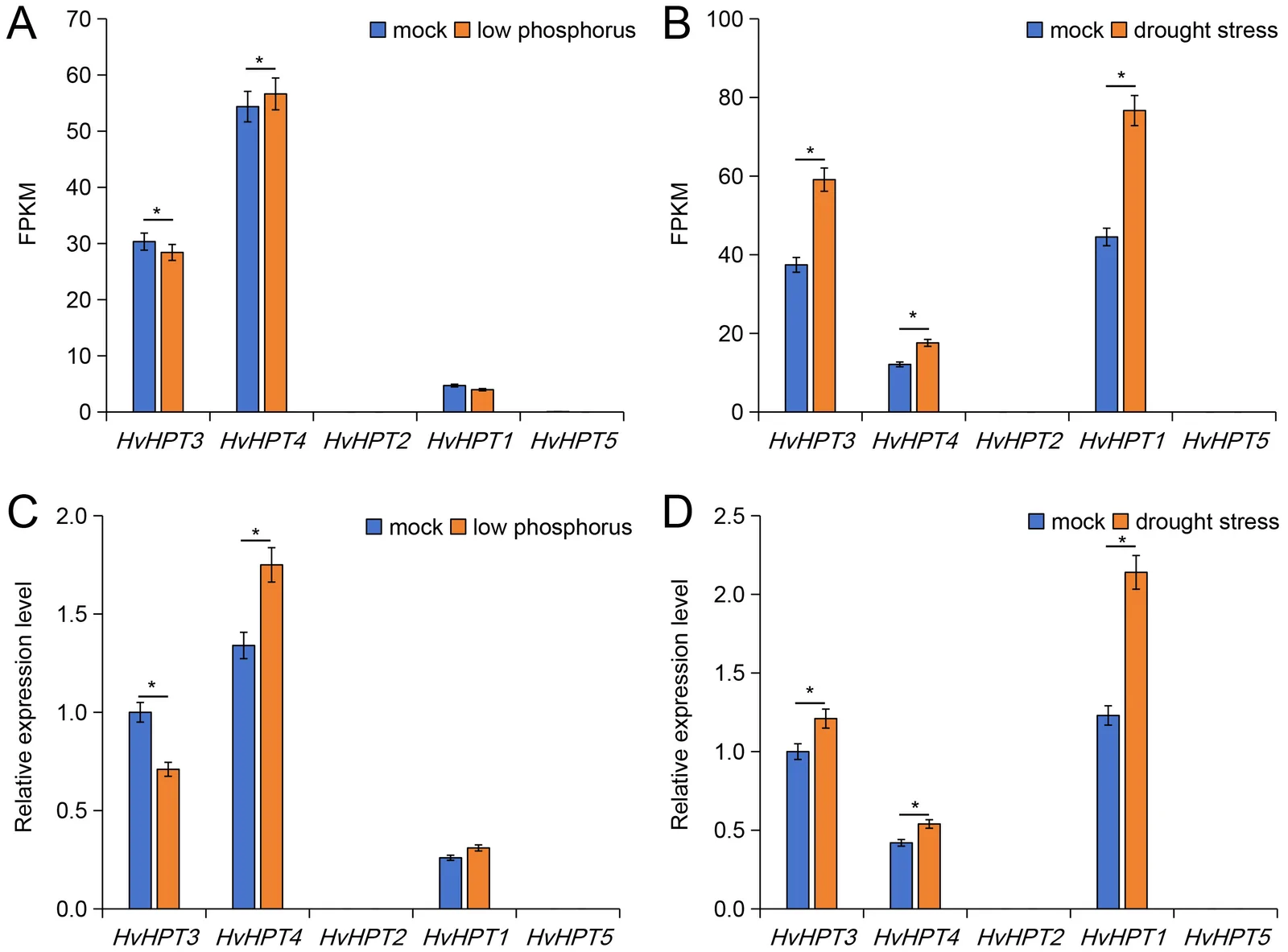
Expression profiles of *HvHPT1-HvHPT5* under low-phosphorus and drought stresses determined by RNA-seq and RT-qPCR. Three independent biological replicates were performed for each treatment group. Error bars represent standard error of the mean (SEM). Two-group comparisons (mock vs stress) were performed using independent-samples t-test; asterisks denote significant differences (*P < 0.05). **(A, B)** Transcript abundance (FPKM values) of five HvHPT members under low-phosphorus **(A)** and drought **(B)** treatments from RNA-seq data. **(C, D)** Relative expression levels validated by RT-qPCR under low-phosphorus **(C)** and drought **(D)** treatments. Mock represents the untreated control group.

## Discussion

Low-phosphorus stress is one of the main abiotic stresses affecting crop growth. Plants activate stress response mechanisms through gene expression regulation, and the response patterns of different tissues have significant spatiotemporal specificity (Nasr Esfahani et al., 2025; Zheng et al., 2024). Previous studies by our team found that the response of barley leaves to low-phosphorus stress showed a time-dependent gene expression activation characteristic: only 154 differentially expressed genes were screened on the 3rd day of low-phosphorus stress, 1990 DEGs on the 19th day of sustained low-phosphorus starvation, and 2686 DEGs at day 23 following Pi-resupply treatment rather than continued low-phosphorus stress. This indicated that under short-term low-phosphorus stress, barley leaves only activated the basic stress perception and response pathways; under Pi-resupply condition, they realized the reprogramming of physiological metabolism through the expression changes of a large number of genes at the transcriptional level to adapt to the growth environment with phosphorus limitation. This characteristic is similar to the response law of tomato leaves to low-phosphorus stress (Satheesh et al., 2022), in which the number of differentially expressed genes in tomato leaves increased nearly 5 times on the 7th day of low-phosphorus stress treatment compared with the 3rd day. Transcriptome sequencing studies on rice under low-phosphorus stress also confirmed that the crop response to low-phosphorus stress had the characteristic of delayed activation (Secco et al., 2013).

It is worth noting that the raw sequencing dataset used in this work was originally reported in our earlier full-length transcriptome study (Ren et al., 2018). The original publication adopted traditional differential-expression-gene-oriented transcriptome analysis, comparing transcriptome differences between phosphorus-efficient genotype GN121 and phosphorus-ensitive genotypes GN42 genotypes, and uncovered large numbers of stress-responsive DEGs participating in phosphorus transportation, phospholipid degradation, galactolipid substitution, SPX-family transcription factor regulation, organic-acid secretion and hormone signal transduction, establishing a general conceptual model for barley phosphorus-starvation acclimatization. Nevertheless, the conventional DEG-mining strategy in the 2018 study could not further prioritize key hub genes tightly coupled with physiological traits such as leaf phosphorus concentration, and it did not dissect gene-to-gene co-regulatory relationships. Moreover, genes related to vitamin E biosynthesis were merely scattered in large DEG datasets without targeted exploration of their potential biological function under phosphorus deficiency. Therefore, further re-analysis is required to dig deeper biological information hidden in the original multi-time-point leaf transcriptome data of the phosphorus-efficient barley GN121.

Weighted Gene Co-expression Network Analysis (WGCNA) can effectively divide differentially expressed genes into functional modules and screen key hub genes closely associated with traits by mining the co-expression relationships among genes, and it has become an important tool for analyzing the molecular regulatory network of crop stress (Barratt et al., 2023). In this study, 4043 differentially expressed genes under low-phosphorus stress were divided into 14 co-expression modules, among which the MEbrown and MEred modules were highly significantly correlated with leaf phosphorus content, being the key modules of barley leaves in response to low-phosphorus stress. In this study, the gene *HvHPT4* (HORVU.MOREX.r3.1HG0080260) with the highest connectivity and gene significance was screened from the MEbrown module positively correlated with phosphorus content. This gene encodes homogentisate phytyltransferase, which can play a role in antioxidant protection and stress response regulation under abiotic stresses such as water deficit, temperature change, light stress and mechanical damage by regulating tocopherol synthesis (Abbasi et al., 2007; Ren et al., 2011; Upadhyaya et al., 2021). Homogentisate phytyltransferase (HPT) is a key rate-limiting enzyme in the plant vitamin E biosynthetic pathway, catalyzing the condensation reaction of homogentisic acid and geranylgeranyl pyrophosphate. Its encoding gene plays an important role in plant stress resistance, growth and development (Ji et al., 2016).

Notably, *HvHPT4* was identified as a DEG in the original 2018 transcriptome dataset, but its biological significance under low-phosphorus stress had never been characterized before. In this work, we performed the first genome-wide identification of barley *HPT* gene family and systematically characterized five family members (*HvHPT1~HvHPT5*), including their chromosomal distribution, protein physicochemical features, gene-exon-intron structures, conserved motifs, and promoter cis-acting regulatory elements. qRT-PCR validation confirmed that *HvHPT3* and *HvHPT4* are responsive to low-phosphorus and drought stress. Other family members exhibit stress-specific or negligible expression under the tested conditions. These observations establish a novel functional link between tocopherol/vitamin E biosynthesis pathway and leaf low-phosphorus tolerance in barley, which constitutes new biological insight not obtained in the original study (Ren et al., 2018).

This study identified 5 members of the HPT gene family in barley for the first time at the genome-wide level, named HvHPT1~HvHPT5. Among them, HvHPT3 and HvHPT4 were localized on chromosome 1H, and HvHPT1 and HvHPT5 on chromosome 7H. Conserved motif analysis showed that Motif 1~2 were the core conserved motifs of the barley HPT family and the key parts constituting the catalytic domain of HPT proteins. Motif 7~8 only existed in HvHPT2 and HvHPT1, which might be an important structural basis for the functional differentiation of HPT genes. Such specific motifs might cause differences in catalytic efficiency and substrate binding specificity among different HPT proteins by affecting the spatial structure of proteins. Analysis of promoter cis-acting elements is an important means to analyze the gene expression regulation pattern. This study found that the promoter regions of barley HvHPT genes contained a large number of abiotic stress response elements, hormone response elements and light response elements. Among them, the number of abiotic stress response elements in the promoter region of HvHPT3 was significantly more than that of other members, including various elements such as drought response and defense response elements, which was highly consistent with the up-regulation expression characteristic of HvHPT3 under low-phosphorus stress, osmotic stress, drought stress and zinc stress. Cis-acting element prediction via PlantCARE provides sequence-level hints for transcriptional regulation, yet these in-silico predictions alone cannot confirm actual biological regulatory function. In the present study, we extracted the 2000 bp upstream promoter regions of five *HvHPT* genes and cataloged putative stress- and hormone-responsive motifs. It should be emphasized that we did not conduct formal statistical enrichment analysis against the genome-wide promoter background of barley; therefore, whether these observed motifs are significantly enriched relative to the genomic background remains to be verified by further large-scale computational or experimental assays such as ChIP-seq. Regarding promoter evolutionary conservation, paralogous *HvHPT* members exhibit substantial divergence in promoter architecture, despite relatively conserved coding-region protein domains. Even for phylogenetically close *HvHPT3* and *HvHPT4* located on chromosome 1H, their promoter cis-element repertoires differ markedly, implying that promoter divergence is a major driver of functional differentiation among barley HPT paralogs. We further compared promoter element composition with our RNA-seq and RT-qPCR expression profiles. *HvHPT3* possesses abundant drought- and general defense-response cis-elements in its promoter, which is consistent with its up-regulation under low-phosphorus and drought stress validated by our RT-qPCR, as well as reported expression changes under osmotic and zinc stress according to public BarleyExpDB resources (not experimentally verified in the present study). *HvHPT4* contains hormone-related cis-elements (abscisic acid, MeJA-responsive motifs), matching its significant induction under both low-phosphorus and drought stress. By contrast, *HvHPT2* and *HvHPT5*, which showed almost no detectable expression under tested stress treatments, lack abundant stress-responsive cis-regulatory motifs in their promoter sequences. This correspondence supports that promoter composition partly shapes the stress-dependent expression behavior of *HvHPT* family members. We manually screened for well-known phosphorus-stress-responsive cis-motifs, including PHR1-binding site (P1BS), WRKY, MYB, bHLH and ABI4-related elements. Scattered copies of MYB, bHLH and WRKY binding sites could be detected across several *HvHPT* promoters; however, canonical P1BS (PHR1-binding) motifs were absent in all five *HvHPT* 2000 bp promoter regions, and no obvious enrichment of phosphorus-specific responsive elements was observed. This suggests that transcriptional activation of *HvHPT3*/*HvHPT4* under low-phosphorus conditions is likely indirect, possibly mediated by other upstream stress-hormone signaling cascades rather than direct binding of PHR-family central phosphorus-regulators to their promoters. Nevertheless, all above-mentioned inferences are based solely on *in-silico* prediction. Future work including promoter-GUS reporter assays and transcription-factor binding validation will be required to validate these predicted regulatory interactions. It should be emphasized that *HvHPT4* was screened as a hub gene solely based on topological connectivity metrics from the WGCNA co-expression network. WGCNA captures statistical co-expression associations and cannot establish causal regulatory relationships; accordingly, *HvHPT4* should be treated as a candidate hub rather than a confirmed master regulator. In the absence of protein-protein interaction prediction, formal regulatory-network inference, and genetic evidence from knockout, overexpression or mutant lines, we cannot definitively conclude that *HvHPT4* functions as a master regulator of low-phosphorus responses. To provide additional correlative bioinformatic support, we performed GO and KEGG functional enrichment for genes tightly co-expressed with *HvHPT4* within the MEbrown module. Neighbouring co-expressed genes were significantly enriched in functional terms associated with reactive oxygen species scavenging, vitamin E biosynthesis, chloroplast membrane homeostasis, abiotic stress response, phosphorus metabolic process and signal transduction. This enrichment pattern is biologically consistent with *HvHPT4*’s annotated function in tocopherol biosynthesis and its observed induction under low-phosphorus stress, offering indirect supporting evidence for its potential involvement in leaf low-phosphorus adaptation. Nevertheless, this evidence remains purely correlative. Future complementary work including protein-interaction prediction, transcription-factor-centred regulatory-network inference, and genetic experiments using CRISPR-Cas9 knockout, gene overexpression or barley mutant materials will be required to test whether HvHPT4 is indeed a causal master regulator, or merely a stress-responsive downstream effector gene within the low-phosphorus transcriptional cascade. Previous studies have confirmed that HPT genes are involved in the response of Arabidopsis to light stress (Collakova and DellaPenna, 2003), rice to salt stress and drought stress (Chen et al., 2016), and potato to heavy metal stress and other abiotic stresses (Upadhyaya et al., 2021). This study found for the first time that HvHPT4 was significantly up-regulated under low-phosphorus stress and was the key hub gene of barley leaves in response to low-phosphorus stress, which greatly expanded the range of stress resistance functions of HPT genes. As a key enzyme for vitamin E synthesis, the up-regulation of HvHPT can promote the biosynthesis of vitamin E in leaves. Vitamin E is not only an important fat-soluble antioxidant that can directly scavenge reactive oxygen species and protect chloroplast membrane structure, but also can enhance plant stress tolerance by regulating the activity of antioxidant enzymes and participating in signal transduction. Therefore, it is hypothesized that up-regulated *HvHPT4* under low-phosphorus stress may contribute to barley leaf low-phosphorus adaptation potentially via vitamin-E-dependent antioxidant defence. Nevertheless, this mechanistic interpretation remains speculative, because we have not quantified endogenous tocopherol content, ROS accumulation or antioxidant enzyme activity in barley leaf tissues, and no genetic manipulation (overexpression/knockout/knockdown) of *HvHPT4* was performed in this study. Direct experimental evidence is still required to verify whether *HvHPT4*-mediated vitamin E biosynthesis could truly mediate low-phosphorus tolerance in barley leaves. In addition, there are significant differences in the stress response characteristics of other members of the barley HPT gene family. HvHPT2 and HvHPT5 showed no significant expression changes under various stresses, indicating that functional differentiation occurred in the HPT gene family in barley. It should be emphasized that, given that our analyses are restricted to a single genotype GN121 and lack direct genetic functional validation, HvHPT3 and HvHPT4 should be treated as candidate genes rather than validated breeding targets for phosphorus-efficiency improvement. Their practical application in crop breeding awaits multi-germplasm evaluation and solid functional experimental proof. In the present RT-qPCR validation experiment, we did not measure leaf relative water content or other physiological indicators to quantify the actual severity of drought stress; additionally, the drought treatment via withholding nutrient solution combined water-deficit and nutrient-deprivation effects, which should be considered when interpreting drought-response expression data. Although this study clarified the characteristics and stress resistance of the barley HvHPT gene family, there are still several aspects that need further in-depth research. First, gene function verification experiments such as overexpression and CRISPR/Cas9 gene knockout are needed to clarify the direct regulatory effect of HvHPT genes on low-phosphorus tolerance in barley. Second, it is necessary to further study the molecular mechanism of HvHPT genes in plant response to stress and clarify their specific regulatory pathways in the low-phosphorus stress regulatory network. Third, the stress resistance function of HvHPT genes in different crops should be verified to provide gene resources for cross-species stress resistance genetic improvement.

While this study identifies *HvHPT3* and *HvHPT4* as promising candidates for low-P tolerance, several caveats must be acknowledged when extrapolating these findings to barley breeding. First, all analyses are based on transcriptomic reanalysis, promoter prediction, and controlled-environment experiments with a single genotype (GN121). We do not present evidence that *HvHPT* expression correlates with grain yield or phosphorus-use efficiency (PUE) under field conditions. In wheat and barley, genotype × phosphorus × environment (G × P × E) interactions for P responsiveness are substantial under field conditions, and the relationship between controlled-environment transcriptomic responses and agronomic PUE is often inconsistent (Deng et al., 2018). For instance, a two-year field study showed that two winter wheat cultivars achieved similar yield potential through distinct P acquisition and utilization strategies, highlighting that molecular responses observed under controlled conditions do not always predict field-level P efficiency (Deng et al., 2018). Therefore, the positive correlation between *HvHPT4* expression and leaf P content in GN121 should be treated as a hypothesis requiring validation in multi-location, multi-year field trials that measure grain yield, biomass, and PUE. Second, whether *HvHPT* genes perform similarly across diverse barley germplasm is currently unknown. Natural variation in tocopherol biosynthesis genes is well documented in other cereals: genome-wide association studies in rice have identified significant loci associated with seed tocopherol content (Kazemzadeh et al., 2024), and different haplotypes of *VTE4* in maize exhibit markedly different effects on vitamin E accumulation (Meena et al., 2025). Similarly, quinoa germplasm displays wide genetic diversity for tocopherol content (22.3~121.6 mg/kg) and composition, with high broad-sense heritability (Velasco et al., 2025). Barley germplasm, including Tibetan wild barley and landraces, harbors considerable genetic diversity for low-P adaptation traits such as root plasticity and P remobilization. However, whether superior or inferior alleles of *HvHPT3*/*HvHPT4* exist in these resources, and whether their promoter or coding-sequence polymorphisms alter stress responsiveness, remains to be investigated through GWAS or haplotype analysis. Third, WGCNA identifies hub genes based on co-expression guilt-by-association, and these genes may be correlated responders rather than causal regulators of the target trait. As noted in recent crop transcriptome studies, correlation does not imply causation, and the proposed functions of hub genes remain hypothetical until validated by functional experiments such as gene overexpression, knockout, or silencing (Luo et al., 2026). Moreover, a substantial gap exists between omics discovery and field deployment: many candidate genes identified through transcriptomics are never functionally validated or advanced into elite breeding lines because of the lack of downstream validation pipelines (Iqbal et al., 2026). Consequently, the designation of *HvHPT4* as a key hub gene should be regarded as a discovery-stage outcome that requires CRISPR/Cas9-mediated functional validation and agronomic phenotyping before it can be confidently prioritized as a breeding target.

Another important caveat should be noted: all co-expression network construction and hub-gene mining in this study were performed using only the phosphorus-efficient genotype GN121. At present, it is still uncertain whether the MEbrown module and hub gene *HvHPT4* identified here represent conserved regulatory modules broadly associated with barley phosphorus-use efficiency, or whether they merely reflect genotype-specific transcriptional reprogramming unique to GN121 under low-phosphorus stress. Although leaf transcriptome sequencing data of the phosphorus-sensitive genotype GN42 are available in our public dataset (Ren et al., 2018), parallel WGCNA analysis and hub-gene screening were not conducted for GN42 in the current study. Therefore, we cannot exclude the possibility that the gene responses observed are GN121-specific. Parallel transcriptomic re-analysis and experimental validation using GN42 and broader barley germplasm are needed in follow-up research to distinguish genotype-specific responses from universal phosphorus-efficiency-related molecular mechanisms.

## Data Availability

The original contributions presented in the study are included in the article/**Supplementary Material**. Further inquiries can be directed to the corresponding author.
