# Supplementary material for: Mining core genes for low-phosphorus tolerance in barley leaves based on WGCNA and analysis of the HPT gene family
Source: Front Plant Sci. 2026 Oct 30;17:1935451. doi: 10.3389/fpls.2026.1935451 (PMC13759403; doi:10.3389/fpls.2026.1935451)
Supplement: Supplementary file 1 [file Table5.docx]

Supplementary Material

# 1 Supplementary Figures and Tables

## 1.1 Supplementary Figures

**Supplementary Figure 1.** Soft connectivity and scale free topology determination.

**Supplementary Figure 2.** Gene clustering tree of gene co-expression network. Different colors represent different modules in network.

**Supplementary Figure 3.** Synteny analysis of *HPT* genes between *Hordeum vulgare* (Hv-Chr*)and *Arabidopsis thaliana* (At-Chr*). Red lines rep resent syntenic relationships between chromosomal regions of different species.

**Supplementary Figure 4.** Sequence alignment of *HPT* genes between barley and Arabidopsis.

**Supplementary Figure 5.** Domain analysis of *HvHPT* gene family members.

**Supplementary Figure 6.** Analysis of cis-elements in the promoter of *HvHPT* gene family members.

**Supplementary Figure 7.** Visualization of the MEred module gene-interaction network.

## 1.2 Supplementary Tables

**Supplementary Table 1.** The Primers of qRT-PCR analysis.

**Supplementary Table 2.** Gene list of 14 modules.

**Supplementary Table 3.** Protein properties of *HvHPT* gene family gene.

**Supplementary Table 4.** Top 20 genes in the MEred and MEbrown modules.
